# NSUN3 promotes oral squamous cell carcinoma progression through autophagy activation and FOXO pathway modulation

**DOI:** 10.3389/fonc.2026.1807220

**Published:** 2026-04-22

**Authors:** Chengtao Wang, Lang Cheng, Lei Wang, Tao Luo, Yuxin Xie, Junliang Chen, Shuangjiang Wu

**Affiliations:** 1 Department of Oral and Maxillofacial Surgery, The Affiliated Stomatological Hospital, Southwest Medical University, Luzhou, China; 2 School of Stomatology, Southwest Medical University, Luzhou, China; 3 Oral & Maxillofacial Reconstruction and Regeneration of Luzhou Key Laboratory, The Affiliated Stomatological Hospital, Southwest Medical University, Luzhou, China; 4 Department of Stomatology, Liuzhou People’s Hospital, Liuzhou, China; 5 Department of Oral and Maxillofacial Surgery, The Affiliated Hospital, Southwest Medical University, Luzhou, China; 6 Department of Pathology, The Fifth Hospital of Deyang, Deyang, China; 7 Department of General Dentistry, The Affiliated Stomatological Hospital, Southwest Medical University, Luzhou, China; 8 Department of Aesthetic Medicine, The Affiliated Stomatological Hospital, Southwest Medical University, Luzhou, China

**Keywords:** autophagy, FOXO pathway, NSUN3, oral squamous cell carcinoma, tumor progression

## Abstract

**Background:**

Oral squamous cell carcinoma (OSCC) is a prevalent malignancy with high rates of lymph node metastasis and recurrence, contributing to persistently poor clinical outcomes. RNA 5-methylcytosine (m^5^C) modification, mediated by methyltransferases such as NSUN3, is implicated in tumor progression; however, the specific function of NSUN3 in OSCC remains largely unexplored. Autophagy plays a dual role in cancer, and the FOXO pathway is a key regulator of autophagy. This study aimed to elucidate the function of NSUN3 in OSCC and its potential mechanism involving autophagy and FOXO signaling.

**Methods:**

NSUN3 expression profiles were characterized in OSCC tissues and cell lines using immunohistochemistry, qRT−PCR, and Western blot. Clinicopathological correlations and survival analyses were performed on a cohort of 60 OSCC patients. NSUN3 was knocked down in SCC15 and SCC25 cells using lentiviral shRNA. Cell proliferation, migration, and invasion were assessed by CCK-8, wound-healing, and Transwell assays. Autophagy activity was evaluated by immunofluorescence (LC3 puncta formation), electron microscopy (autophagosome quantification), and Western blot (LC3-II/I ratio, Beclin1, P62/SQSTM1). The autophagy agonist rapamycin was used to rescue phenotypic changes. The activity of the FOXO pathway was assessed by detecting phospho-FOXO1/FOXO3.

**Results:**

NSUN3 was significantly upregulated in OSCC tissues and cells. Elevated NSUN3 expression, along with advanced pTNM stage and lymph node metastasis, constituted independent risk factors for poor overall survival. NSUN3 knockdown suppressed OSCC cell proliferation, migration, and invasion. Mechanistically, NSUN3 depletion inhibited autophagy, as evidenced by reduced LC3 puncta, decreased autophagosome number, lower Beclin1 expression, a reduced LC3-II/I ratio, and increased P62/SQSTM1 levels. Rapamycin treatment reversed these effects and partially restored malignant phenotypes. Furthermore, NSUN3 knockdown increased the phosphorylation (inactivation) of FOXO1 and FOXO3, thereby suppressing the FOXO signaling pathway.

**Conclusion:**

NSUN3 is overexpressed in OSCC and is an independent prognostic factor. It promotes OSCC progression by enhancing autophagy, potentially through modulating the FOXO pathway. Targeting NSUN3 may represent a novel therapeutic strategy for OSCC.

## 1 Introduction

OSCC accounted for more than 350, 000 new cases worldwide in 2022, making it the 16th most frequently diagnosed cancer. The five-year survival rate for OSCC has remained stagnant at approximately 50-60% (1, 2). This poor prognosis is largely driven by a high propensity for lymph node metastasis (20-40% even in early-stage patients) and postoperative recurrence (>40%) (3). Despite advances in multimodal therapies, the lack of effective targeted strategies underscores the urgent need to elucidate the molecular mechanisms driving OSCC progression.

In the field of epigenetics, RNA m^5^C methylation has garnered significant attention. Dysregulation of m^5^C modification has been implicated in the pathogenesis and progression of various human diseases, including cancer. This dynamic process is orchestrated by m^5^C methyltransferases (“writers”), demethylases (“erasers”), and m^5^C-binding proteins (“readers”). The RNA m^5^C methyltransferase family primarily comprises the NOL1/NOP2/sun (NSUN) methyltransferases and the DNA methyltransferase homolog DNMT2 (4–7). Among them, NSUN3 is predominantly localized to the mitochondrial matrix in human cells, and its deficiency precipitates mitochondrial dysfunction (8, 9). In head and neck squamous cell carcinoma (HNSCC), NSUN3 functions as an oncogenic driver, accelerating cancer cell proliferation and tumorigenicity. Notably, NSUN3 expression is inversely correlated with tumor-infiltrating immune cells, with lower NSUN3 levels associated with higher immune scores in HNSCC patients (10). Furthermore, elevated NSUN3 expression predicts lymph node metastasis and poor patient prognosis (11). In hepatocellular carcinoma, NSUN3 participates in modulating the tumor immune microenvironment (12). In lung squamous cell carcinoma, NSUN3 mRNA is significantly upregulated compared to normal tissues and closely correlates with clinicopathological features (13). Studies also indicate that NSUN3 regulates the metabolic reprogramming necessary for primary tumor cell invasion and dissemination via mitochondrial RNA modification (14). However, the specific biological functions and underlying mechanisms of NSUN3 in OSCC remain largely unexplored.

Autophagy is a self-degradative process that removes damaged organelles, misfolded proteins, and other intracellular components via lysosomal degradation (15). In cancer biology, autophagy plays a dual role (16). It can function as a tumor suppressor by degrading oncogenic proteins, such as SRC, thereby inhibiting metastasis (17). Conversely, it can support tumor cell survival by facilitating metabolic adaptation (18). For example, autophagy induction inhibits cancer cell proliferation in colorectal cancer (19). However, in endometrial and ovarian cancers, tumor cells exhibit heightened autophagic activity. Autophagy can provide nutrients for tumor cells, promoting their proliferation and resistance (20, 21).

The Forkhead box class O (FOXO) family of transcription factors plays pivotal roles in regulating diverse physiological processes. The FOXO signaling pathway is involved in controlling the cell cycle, apoptosis, autophagy, tumor suppression, and metabolism. Specifically, FOXO3a is a core regulator of autophagy and can directly transactivate key autophagy-related genes such as LC3 and Beclin1 (22). Furthermore, other RNA modifications (e.g., m^6^A) have been shown to regulate autophagy via FOXO3 (23).

Consequently, this study aims to investigate the role of NSUN3 in OSCC progression. We seek to determine whether NSUN3 regulates the FOXO pathway and examine the extent to which autophagy mediates this regulatory axis. Our findings offer novel insights that could inform the development of targeted therapeutic strategies for OSCC.

## 2 Materials and methods

### 2.1 Bioinformatics analysis

Given the absence of a publicly available transcriptomic dataset specific to OSCC, we performed initial bioinformatic exploration using the TCGA head and neck squamous cell carcinoma (HNSC) dataset, which includes OSCC as a major subgroup. RNA-seq data and corresponding clinical information for HNSC were retrieved from The Cancer Genome Atlas (TCGA) database, comprising a total of 567 samples. Gene set enrichment analysis (GSEA) for NSUN3 was subsequently conducted using R software (version 4.4.1) with relevant Bioconductor packages to identify potentially associated pathways. To ensure OSCC-specificity, all bioinformatic predictions were subsequently validated through functional assays in OSCC cell lines and clinical correlation in an independent OSCC patient cohort.

### 2.2 Clinical samples

Sixty primary OSCC patients that underwent surgical resection in the Department of Oral and Maxillofacial Surgery, the Affiliated Stomatology Hospital of Southwest Medical University were enrolled in this study. Written informed consent was obtained from all patients. All patients underwent radical resection with safe surgical margins (≥2 cm), neck lymph node dissection, and flap reconstruction, based on tumor size, between January 2018 and September 2023. Inclusion criteria comprised patients with complete clinical and follow-up data, a confirmed histopathological diagnosis of OSCC, no prior neoadjuvant therapy, such as immunotherapy or transcatheter arterial chemoembolization, and availability of intact paraffin-embedded specimens. The Ethics Committee of the Affiliated Stomatology Hospital, Southwest Medical University authorized the research.

### 2.3 Cell lines and culture

The human oral keratinocyte (HOK) line and three OSCC lines (SCC9, SCC15, SCC25) were obtained from the Cell Bank of the Chinese Academy of Medical Sciences. Cells were maintained in DMEM/F12 medium supplemented with 10% fetal bovine serum (FBS) at 37 °C in a humidified atmosphere containing 5% CO_2_. The culture medium was replaced every two days, and cells were subcultured upon reaching 80% confluence. Rapamycin (Rap) was purchased from TargetMol (#T1537). For induction of autophagy signaling, cells were cultured in medium supplemented with 100 nM Rap for 24 hours.

### 2.4 Quantitative real-time PCR

Total RNA was extracted from the indicated cell lines using a commercial kit, followed by cDNA synthesis using a reverse transcription reagent kit. The mRNA expression level of NSUN3 was quantified by qRT-PCR using a SYBR Green-based master mix. GAPDH served as the endogenous control, and relative expression was calculated using the 2−ΔΔCt method.

### 2.5 Western blotting

Cells were lysed in RIPA buffer, and total protein concentration was quantified using a BCA assay. Equal amounts of protein were separated by SDS-PAGE and transferred onto PVDF membranes. After blocking, the membranes were incubated with primary and HRP-conjugated secondary antibodies sequentially. Protein bands were visualized using an enhanced chemiluminescence (ECL) substrate. The primary antibodies used included NSUN3(1:2500, Abcam, #ab272616), LC3B (1:1000, Abmart, #T55992), P62/SQSTM1 (1:8000, Abmart, #T55546), Beclin1(1:2000, Abmart, #T55092), FOXO1 Ab(1:1000, Abways, #CY7234), FOXO3a(1:1000, Abways, #CY5898), p-FOXO1Ab(1:500, Abways, #CY6217), p-FOXO3a(1:2000, HUABIO, #ET1609-49). GAPDH (1:5000, Proteintech, #10494-1-AP) was selected as the internal reference gene.

### 2.6 Lentiviral transduction and stable cell line generation

Three shRNA sequences targeting NSUN3 were designed and synthesized by ZFdows Bio Co., Ltd (Nanjing, China), and subsequently packaged into lentiviral vectors. OSCC cells transduced with these lentiviruses were selected with puromycin for one week. The knockdown efficiency was then evaluated by Western blot analysis and quantitative real-time PCR (qRT-PCR).

### 2.7 Immunofluorescence

Cells were fixed in 4% PFA, permeabilized using 0.3% Triton X-100, and blocked with 5% bovine serum albumin (BSA) for 60 min. After that, cells were incubated with anti-LC3B antibody (Proteintech, #14600-1-AP) overnight at 4 °C, fluorescently labeled secondary antibody against rabbit IgG for 1 h and DAPI for 10 min, respectively. The results were analyzed using a fluorescence microscope.

### 2.8 Immunohistochemistry

Serial 5 μm tissue sections were mounted, deparaffinized, and rehydrated. Antigen retrieval was performed via heat-induced epitope retrieval (HIER) in citrate buffer. Endogenous peroxidase was quenched with 3% H_2_O_2_, and nonspecific sites were blocked with normal serum. Sections were incubated overnight at 4°C with a rabbit anti-NSUN3 primary antibody (Abcam, #ab272616) at a 1:100 dilution. For negative controls, serial sections of adjacent normal oral mucosa were incubated with PBS instead of the primary antibody. An anti-Ki-67 antibody (Abcam, # ab15580) was used as a positive control on adjacent normal oral mucosa to validate the immunostaining procedure. Representative images of these controls are provided in **Supplementary Figure 1**. After washing, an HRP-conjugated goat anti-rabbit secondary antibody was applied. Signal was visualized with DAB, followed by hematoxylin counterstaining, dehydration, and mounting. Digital slides were captured at 400× magnification using a digital slide scanner. The Immunoreactivity Score (IRS) was calculated by multiplying the percentage score of positively stained cells (0–4: 0 = 0%, 1 = ≤25%, 2 = ≤50%, 3 = ≤75%, 4 = ≤100%) by the staining intensity score (0–3: 0 = negative, 1 = weak, 2 = moderate, 3 = strong). All stained sections were evaluated semi−quantitatively by two independent investigators. To minimize recall bias, the same investigators re−scored the images at one−week intervals. Inter−observer reliability, expressed as the kappa value, was 0.970. In case of significant discrepancy between the scores assigned by the two investigators, a third investigator provided the final assessment.

### 2.9 Transmission electron microscopy

Cells were pre-embedded using a preheated and dissolved 1% agarose solution, followed by fixation with 1% osmium tetroxide prepared in 0.1 M phosphate buffer (PB, pH 7.4) for 2 h at room temperature in the dark. Subsequently, the samples were washed and dehydrated through a graded series of ethanol solutions, infiltrated with acetone and SPI 812 embedding medium, and polymerized at 60 °C for 48 h. Ultrathin sections (60–80 nm) were collected on copper grids, double-stained with 2% uranyl acetate in ethanol and 2.6% lead citrate, and examined under a transmission electron microscope (HITACHI, HT7800). In transmission electron micrographs, autophagosomes were identified as vesicular structures enclosed by double or multiple membranes, containing morphologically intact cytoplasmic components (e.g., mitochondria, endoplasmic reticulum fragments, or cytosol). Autolysosomes were identified as single-membrane-bound vesicles with heterogeneous electron density and evident degradation of their contents. Only structures with clear double membranes and intact cargo were counted as autophagosomes.

### 2.10 Cell proliferation assay

Cell proliferation was assessed using a Cell Counting Kit-8 (CCK-8) assay. Cells were seeded into 96-well plates at 5, 000 cells per well. After treatment, the CCK-8 reagent was added to each well according to the manufacturer’s instructions, and the absorbance at 450 nm was measured using a microplate reader.

### 2.11 Transwell assay

For the invasion assay, Transwell chambers were pre-coated with Matrigel. Then, 1×10^5^ cells were seeded into the upper chamber, while the lower chamber was filled with medium containing 10% FBS as a chemoattractant. After 36 h of incubation, cells on the lower surface of the membrane were fixed with 4% paraformaldehyde, stained with 0.1% crystal violet, and imaged under a microscope.

### 2.12 Wound-healing assay

Migration was assessed with a standard wound-healing assay. Briefly, a confluent cell monolayer grown in a 6-well plate was scratched with a sterile 200-µL pipette tip to create a linear wound. The wounded areas were imaged immediately (0 h) and at 24 h post-scratch under a microscope. The migration rate was quantified by measuring the wound closure percentage at the indicated time points using ImageJ software.

### 2.13 Statistical analysis

Statistical analyses were conducted using SPSS software (version 22.0; IBM Corp, Armonk, NY, USA). Data from at least three independent experiments are presented as the mean ± standard deviation (SD). Differences among multiple groups were evaluated by one−way analysis of variance (ANOVA) followed by Tukey’s post−hoc test. The association between NSUN3 expression and clinicopathological parameters was assessed using the Chi−square test. For comparisons between two groups, the independent−samples t−test was applied.

Survival analyses were conducted as follows: The optimal cutoff value for the NSUN3 Immunoreactivity Score (IRS) was determined from the receiver operating characteristic (ROC) curve by selecting the point closest to the upper−left corner (Youden index). Overall survival was analyzed with the Kaplan–Meier method, and differences between survival curves were compared using the log−rank test. A multivariate Cox proportional hazards regression model was employed to evaluate the independent influence of variables including gender, age, clinicopathological stage, NSUN3 IRS score, and lymph node metastasis on survival outcomes, with results expressed as hazard ratios (HR) and 95% confidence intervals (CI). A two−tailed P value < 0.05 was considered statistically significant.

## 3 Results

### 3.1 NSUN3 is upregulated in OSCC and associated with poor prognosis

**Figure 1:**
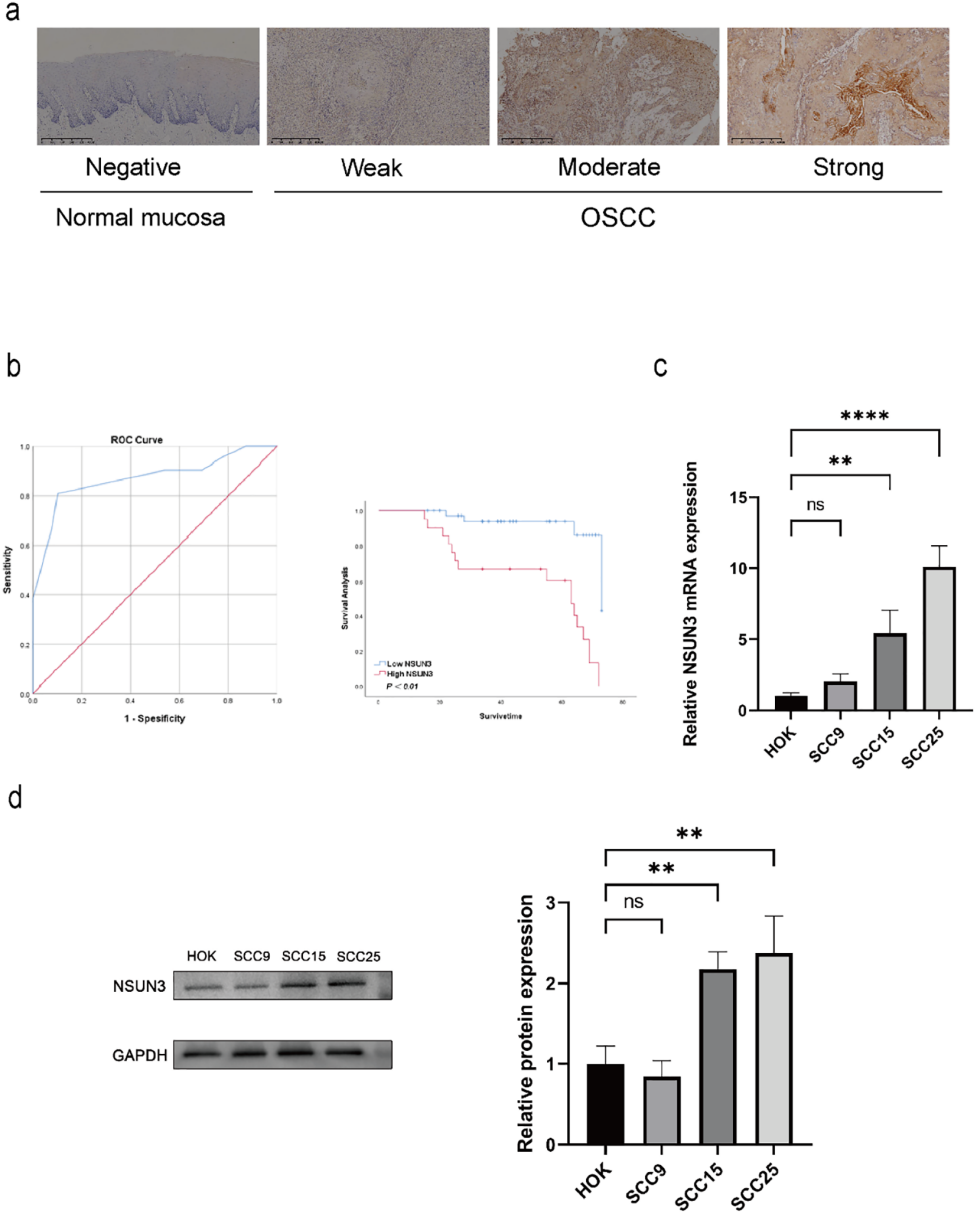
NSUN3 is upregulated in OSCC and associated with poor prognosis. **(A)** Representative immunohistochemical images of various immunostaining (intense, moderate, and weak) of NSUN3 expression in normal oral tissues and OSCC tissues. **(B)** Kaplan–Meier survival curves comparing overall survival between OSCC patients with high vs. low NSUN3 expression, stratified based on the ROC−derived cutoff value (Youden index). Statistical significance was determined using the log−rank test (P < 0.01). **(C)** qRT-PCR for NSUN3 in OSCC cell lines and HOK. **(D)** Western Blot for NSUN3 in OSCC cell lines and HOK. ns: no significant difference; **P < 0.01; ****P < 0.0001.

To investigate the role of NSUN3 in OSCC progression, we first examined its expression in OSCC and normal oral mucosal tissues using immunohistochemistry. NSUN3 immunoreactivity was primarily localized in the cytoplasm. NSUN3 expression was significantly upregulated in OSCC tissues relative to adjacent normal oral mucosa (**Figure 1A**).

Based on the optimal cutoff value derived from ROC analysis (Youden index), OSCC patients were stratified into NSUN3−high and NSUN3−low groups. Kaplan–Meier survival analysis demonstrated that patients with high NSUN3 expression had a significantly poorer overall survival compared to those with low expression (log−rank test, P < 0.01; **Figure 1B**). Multivariate Cox regression analysis (**Table 1**) identified high NSUN3 expression, advanced pTNM stage (III−IV), and lymph node metastasis as independent risk factors for poor overall survival.

**Table 1:** Multivariate analysis of overall survival in OSCC patients (sample size = 60).

		NSUN3 overall survival	
Factor	Subgroup	HR (95%CI)	*P*-value
Gender	Male	1	
	Female	0.478 (0.159-1.437)	0.189
Age	<60 years	1	
	≥60 years	1.623 (0.666-3.956)	0.287
pTNM stage ^a^	I-II	1	
	III-IV	2.297 (0.888-5.938)	0.043 *
NSUN3	Low (IRS<7)	1	
	High (IRS≥7)	10.423 (3.049-35.631)	<0.001 *
Lymph node metastasis	Yes	1	
	No	0.806 (0.325-1.995)	0.039 *

*P < 0.05, statistically significant.

^a^ pTNM stage is classified according to the 8th Edition of the UICC TNM Classification of Malignant Tumors, based on the WHO system.

Consistently, qRT-PCR and Western blotting confirmed elevated mRNA and protein levels of NSUN3 in OSCC cell lines (SCC15 and SCC25) compared with normal oral mucosal cells (**Figures 1C, D**). These findings suggest that NSUN3 upregulation may play an important role in OSCC progression.

### 3.2 NSUN3 promotes proliferation, migration, and invasion of OSCC cells

**Figure 2:**
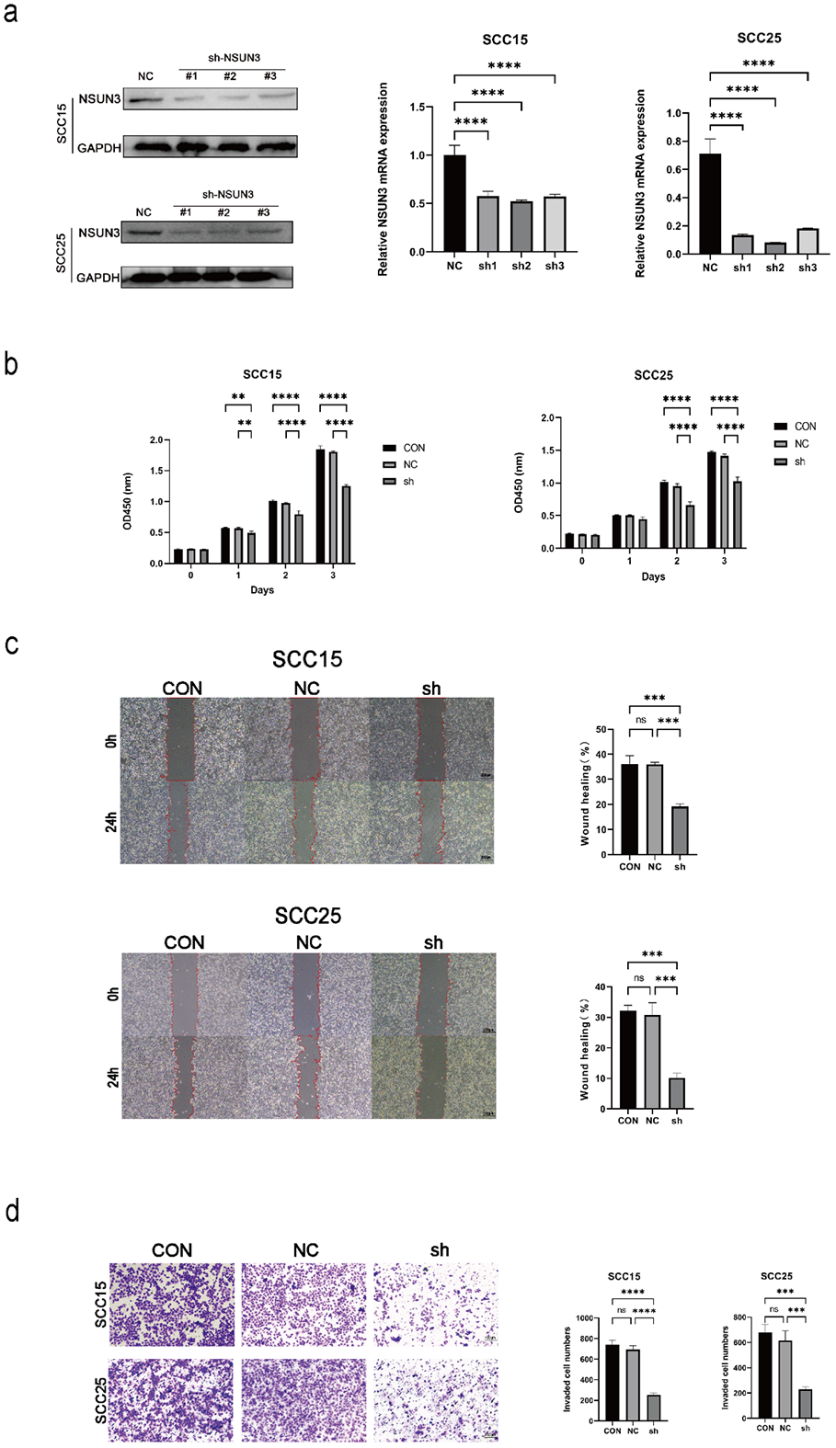
NSUN3 promotes proliferation, migration, and invasion of OSCC cells. **(A)** The protein expression and mRNA levels of NSUN3 in SCC15 and SCC25 cells were determined using western blot and qRT-PCR after transfection with sh-NSUN3. **(B)** CCK-8 assay to determine the inhibitory effect of NSUN3 knockdown on OSCC cell proliferation. **(C)** Wound healing assay demonstrated reduced migratory capacity after NSUN3 knockdown. **(D)** Transwell invasion assay revealed decreased invasive potential following NSUN3 knockdown. ns, no significant difference; **P < 0.01; ***P < 0.001; ****P < 0.0001.

To explore the functional role of NSUN3 in OSCC, we knocked down NSUN3 expression in SCC15 and SCC25 cells using lentivirus−mediated shRNA. The knockdown efficiency was validated at both the mRNA and protein levels (**Figure 2A**), and sh−NSUN3#2 demonstrated the highest potency for subsequent experiments. Accordingly, NSUN3 depletion markedly attenuated cell proliferation, migration, and invasion (**Figures 2B–D**). These results indicate that NSUN3 exerts oncogenic effects in OSCC.

### 3.3 NSUN3 enhances autophagy in OSCC cells

**Figure 3:**
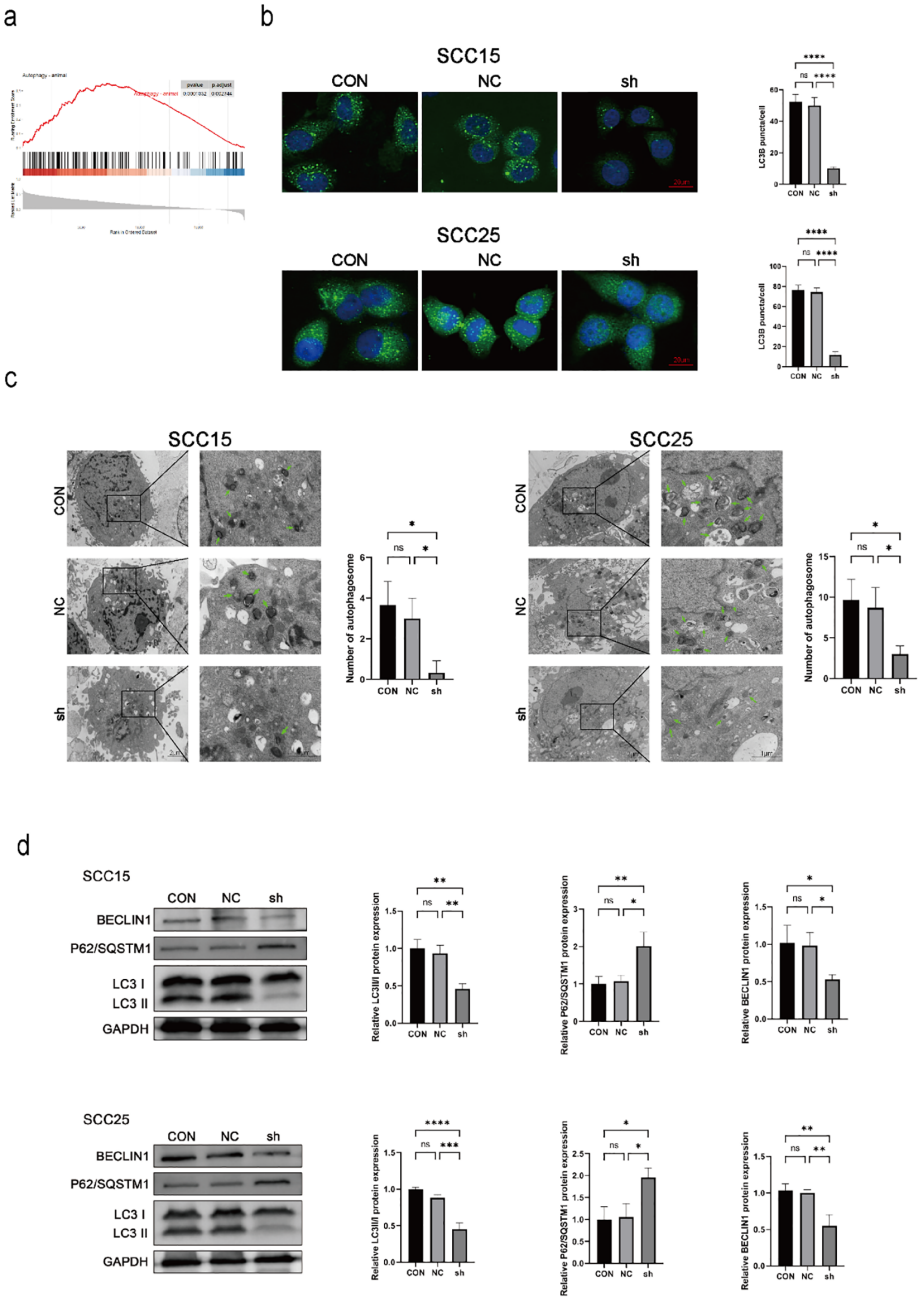
NSUN3 enhances autophagy in OSCC cells. **(A)** Gene Set Enrichment Analysis showed a significant positive correlation between NSUN3 expression and Autophagy in HNSCC. **(B)** Immunofluorescence showed that the number of LC3 puncta in OSCC cells after transfection with sh-NSUN3 decreased. **(C)** Transmission electron microscopy revealed that the number of autophagosomes in OSCC cells transfected with sh-NSUN3 decreased. **(D)** The protein expression levels of Beclin1, P62/SQSTM1, and LC3 in OSCC cells after transfection with sh-NSUN3 were measured using western blot. ns, no significant difference; *P < 0.05; **P < 0.01; ***P < 0.001; ****P < 0.0001.

GSEA demonstrated that NSUN3 expression positively correlated with the autophagy gene set in HNSCC (**Figure 3A**). Following NSUN3 knockdown, LC3 puncta formation was markedly reduced (**Figure 3B**), and the number of autophagosomes was significantly decreased (**Figure 3C**). Western blot analysis showed that Beclin1 was downregulated, accompanied by a reduction in the LC3-II/LC3-I ratio and a concomitant accumulation of P62/SQSTM1 (**Figure 3D**). Thus, NSUN3 promotes autophagy in OSCC cells.

### 3.4 NSUN3 promotes OSCC cell proliferation, migration, and invasion by inducing autophagy

**Figure 4:**
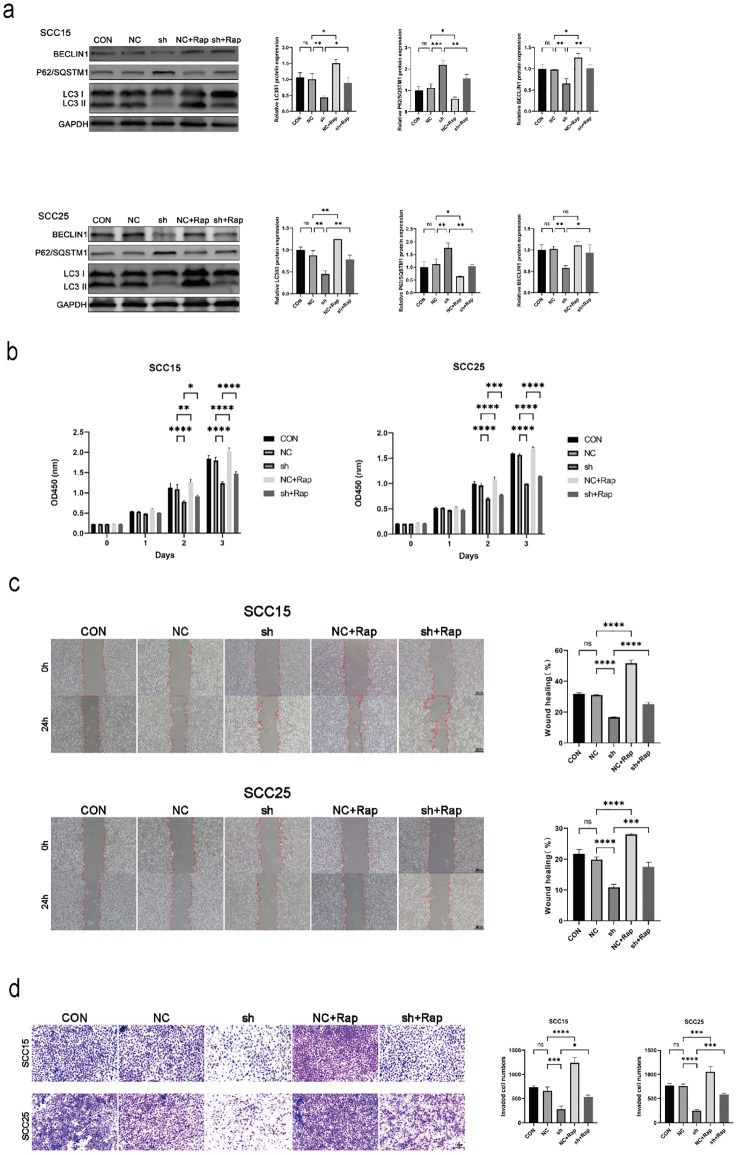
NSUN3 promotes OSCC cell proliferation, migration, and invasion by inducing autophagy. **(A)** The protein expression levels of Beclin1, P62/SQSTM1, and LC3 in OSCC cells after transfection with sh-NSUN3 and treated with autophagy agonist Rap were measured using western blot. **(B)** The proliferation ability of OSCC cells after transfection with sh-NSUN3 and treated with Rap was assessed using the CCK-8 assay. **(C)** The migratory capacity of OSCC cells after transfection with sh-NSUN3 and treated with Rap was assessed using the Wound healing assay. **(D)** The invasion ability of OSCC cells after transfection with sh-NSUN3 and treated with Rap was assessed using the Transwell invasion assay. ns, no significant difference; *P < 0.05; **P < 0.01; ***P < 0.001; ****P < 0.0001.

To determine whether autophagy mediates the aggressive phenotype, NSUN3-deficient SCC15 and SCC25 cells were treated with rapamycin, a potent autophagy agonist. NSUN3 silencing downregulated Beclin1 and the LC3−II/LC3−I ratio but upregulated P62/SQSTM1; these effects were partially ablated or rescued by rapamycin treatment (**Figure 4A**). Furthermore, rapamycin partially rescued the impaired cell proliferation, migration, and invasion resulting from NSUN3 knockdown (**Figures 4B–D**). Collectively, these data indicate that NSUN3 promotes the malignant progression of OSCC, at least in part, by inducing autophagy.

### 3.5 NSUN3 modulates FOXO signaling activity in OSCC cells

**Figure 5:**
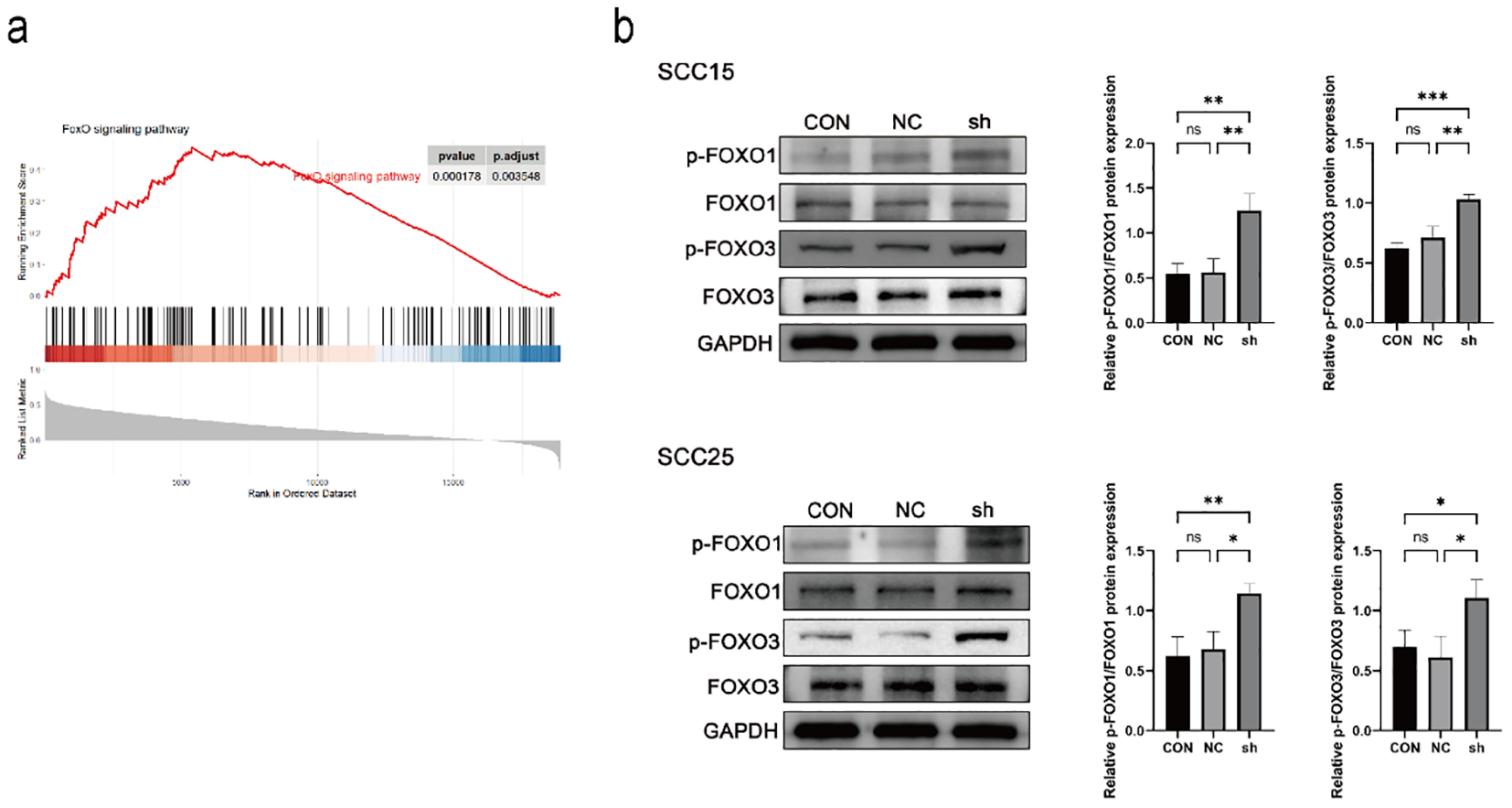
NSUN3 modulates FOXO signaling activity in OSCC cells. **(A)** Gene Set Enrichment Analysis showed a significant positive correlation between NSUN3 expression and the FOXO signaling pathway in HNSCC. **(B)** The protein levels of p-FOXO1, FOXO1, p-FOXO3, and FOXO3 in OSCC cells after transfection with sh-NSUN3 were assessed using western blot. ns, no significant difference; *P < 0.05; **P < 0.01; ***P < 0.001.

GSEA analysis revealed a positive association between NSUN3 expression and FOXO signaling activity in HNSCC (**Figure 5A**). Given the established role of FOXO transcription factors in autophagy regulation, we next investigated whether NSUN3 influences this pathway. Knockdown of NSUN3 significantly increased the phosphorylation levels of FOXO1 and FOXO3, whereas total FOXO1/3 levels remained unaffected (**Figure 5B**). These results indicate that NSUN3 depletion is associated with increased inhibitory phosphorylation of FOXO proteins, suggesting a potential link between NSUN3 and the regulation of FOXO pathway activity.

## 4 Discussion

This study establishes that NSUN3 is significantly overexpressed in OSCC tissues and cell lines. Multivariate analysis validated that its elevated expression, along with advanced TNM stage and lymph node metastasis, constitutes an independent prognostic risk factor in OSCC. Functionally, NSUN3 knockdown markedly inhibited OSCC cell proliferation, migration, and invasion, supporting its oncogenic properties. Importantly, we provide the first evidence that NSUN3 promotes autophagy in OSCC cells, as evidenced by reduced LC3 puncta formation, decreased autophagosome counts, and the modulation of key autophagic markers, specifically the downregulation of Beclin1 and LC3-II/LC3-I alongside P62 accumulation, following NSUN3 silencing. Furthermore, rescue experiments using the autophagy agonist rapamycin partially restored the malignant phenotypes impaired by NSUN3 knockdown, suggesting that the activation of autophagy is a pivotal mechanism through which NSUN3 exerts its pro-tumorigenic effects. Finally, we found that knockdown of NSUN3 increased the phosphorylation levels of FOXO1 and FOXO3, suggesting the potential involvement of the FOXO pathway in this process.

Methylation, a key epigenetic mechanism involving SAM-dependent methyl group transfer to specific substrates, significantly contributes to tumorigenesis (24). RNA methylation encompasses several chemically distinct modifications such as m^6^A, m¹A, m^5^C, and m^7^G (25–27). Among these, m^5^C is widely distributed in human mRNA, rRNA, and tRNA (28, 29). As a reversible epigenetic mark, m^5^C influences RNA stability, protein interactions, and transcription, and is critically involved in tumor initiation, progression, metastasis, and drug resistance (30, 31). The dynamics of m^5^C are regulated by writers (methyltransferases), erasers (demethylases), and readers (m^5^C-binding proteins) (4). The main RNA m^5^C methyltransferases are the NSUN family and DNMT2 (32). The NSUN family includes seven members, with NSUN3 being consistently linked to tumor development, a finding corroborated by our study.

The oncogenic role of autophagy in established tumors is increasingly recognized, where it supports survival under metabolic stress, facilitates invasion, and promotes therapy resistance (16, 21). Our data place NSUN3 within this pro−tumorigenic autophagic network in OSCC. The rescue of malignant phenotypes upon rapamycin treatment in NSUN3−knockdown cells provides supportive evidence that the tumor−promoting effects of NSUN3 are largely autophagy−dependent. This context is crucial, as autophagy functions as a double-edged sword, exhibiting tissue-specific roles in different malignancies. For instance, while it acts as a tumor suppressor in cervical cancer (e.g., via SNX10) and laryngeal carcinoma (e.g., via CH25H) (33, 34), our results, alongside reports in pancreatic cancer (20), illustrate its role as a tumor promoter in specific contexts. This dichotomy underscores that the functional output of autophagy is determined by upstream regulators and cellular context, with NSUN3 emerging as a critical upstream activator in OSCC. It is noteworthy that rapamycin treatment only partially reversed the phenotypic changes induced by NSUN3 knockdown. This partial rescue suggests that while autophagy activation represents an important downstream effector of NSUN3, it is unlikely to be the sole mechanism. As a mitochondrial RNA methyltransferase, NSUN3 may also influence other cellular processes—such as mitochondrial metabolism, oxidative stress response, or redox homeostasis—that contribute to OSCC progression independently of autophagy. Future studies are warranted to explore these possibilities and fully delineate the functional repertoire of NSUN3 in OSCC.

Mechanistically, we link NSUN3 to the FOXO transcription factor family, master regulators of autophagy and cellular homeostasis (22). FOXO proteins promote autophagy primarily through their transcriptional activity: upon activation (via dephosphorylation), they translocate to the nucleus and induce the expression of key autophagy genes such as LC3 and BECN1 (22). Additionally, FOXO proteins can regulate autophagy through cytoplasmic protein interactions (35, 36) and epigenetic modulation (37). In contrast to this activation model, our observation that NSUN3 knockdown increases FOXO1/3 phosphorylation suggests that NSUN3 functions as a negative regulator of inhibitory FOXO phosphorylation, thereby maintaining the functional pool of active FOXO proteins. Thus, loss of NSUN3 leads to FOXO hyperphosphorylation and consequent pathway inhibition. This aligns with an emerging paradigm where RNA modifications fine−tune signaling pathways—for example, YTHDF3 upregulates FOXO3 translation to induce autophagy (23). However, the precise molecular mechanism by which NSUN3 influences FOXO phosphorylation remains to be elucidated. NSUN3, as an RNA m^5^C methyltransferase primarily localized to mitochondria, could potentially regulate upstream signaling components that control FOXO phosphorylation, but this hypothesis requires direct experimental validation. Further studies, including transcriptome-wide analyses to identify direct RNA targets of NSUN3 and functional assays to delineate the signaling cascade, are needed to establish the mechanistic link. Nonetheless, our current data provide the first evidence linking NSUN3 to FOXO signaling in OSCC, offering a foundation for future mechanistic exploration.

In addition, our findings invite consideration of broader pathophysiological implications. Firstly, given NSUN3’s mitochondrial localization and role in tRNA modification (10, 11), its overexpression in OSCC likely contributes to the metabolic plasticity essential for tumor growth and invasion. Autophagy and mitochondrial metabolism are intimately linked, and NSUN3 may serve as a nexus coordinating these processes to fuel OSCC progression. Secondly, NSUN3 has been implicated in modulating the tumor immune microenvironment in HNSCC and hepatocellular carcinoma (10, 12). While our study focused on cell−autonomous mechanisms, it is plausible that NSUN3−driven autophagy in OSCC cells influences cytokine secretion, antigen presentation, or interactions with immune cells, affecting overall tumor immunogenicity and progression.

Despite these insights, our study has limitations. First, although a correlation between NSUN3 expression and FOXO pathway activity was established, the direct causal link and the precise RNA targets of NSUN3 remain to be identified. Second, the *in vivo* role of NSUN3 in OSCC progression awaits validation using animal models. Third, although multiple complementary approaches (immunofluorescence, electron microscopy, and Western blotting) were used to assess autophagy, autophagic flux was not directly measured using lysosomal inhibitors. Moreover, given that autophagy is regulated by a complex network of multiple genes, future studies should incorporate analysis of additional autophagy-related genes to provide a more comprehensive understanding of the autophagic pathways involved in NSUN3-mediated OSCC progression. Fourth, the relatively small sample size (n = 60) and the lack of a formal power calculation represent limitations of this study. Although the strong effect size for NSUN3 (HR = 10.423; 95% CI 3.049–35.631) suggests adequate power to detect this association, the possibility of insufficient power for smaller effects cannot be excluded. Validation in larger independent cohorts is therefore warranted to confirm the prognostic value of NSUN3.

Additionally, the potential crosstalk between NSUN3−regulated autophagy and other cellular processes, such as immune modulation or metabolic reprogramming, remains unexplored. Given that NSUN3 has been linked to immune infiltration in HNSCC and LIHC (10, 12), it is plausible that its effects in OSCC may also involve the tumor microenvironment. Future studies should employ chromatin immunoprecipitation (ChIP) or RNA immunoprecipitation (RIP) assays to examine whether NSUN3 directly influences FOXO target gene expression or modifies relevant RNAs. In addition, the functional significance of the NSUN3−FOXO−autophagy axis should be tested in orthotopic or patient−derived xenograft models to evaluate its therapeutic potential.

**Figure 6:**
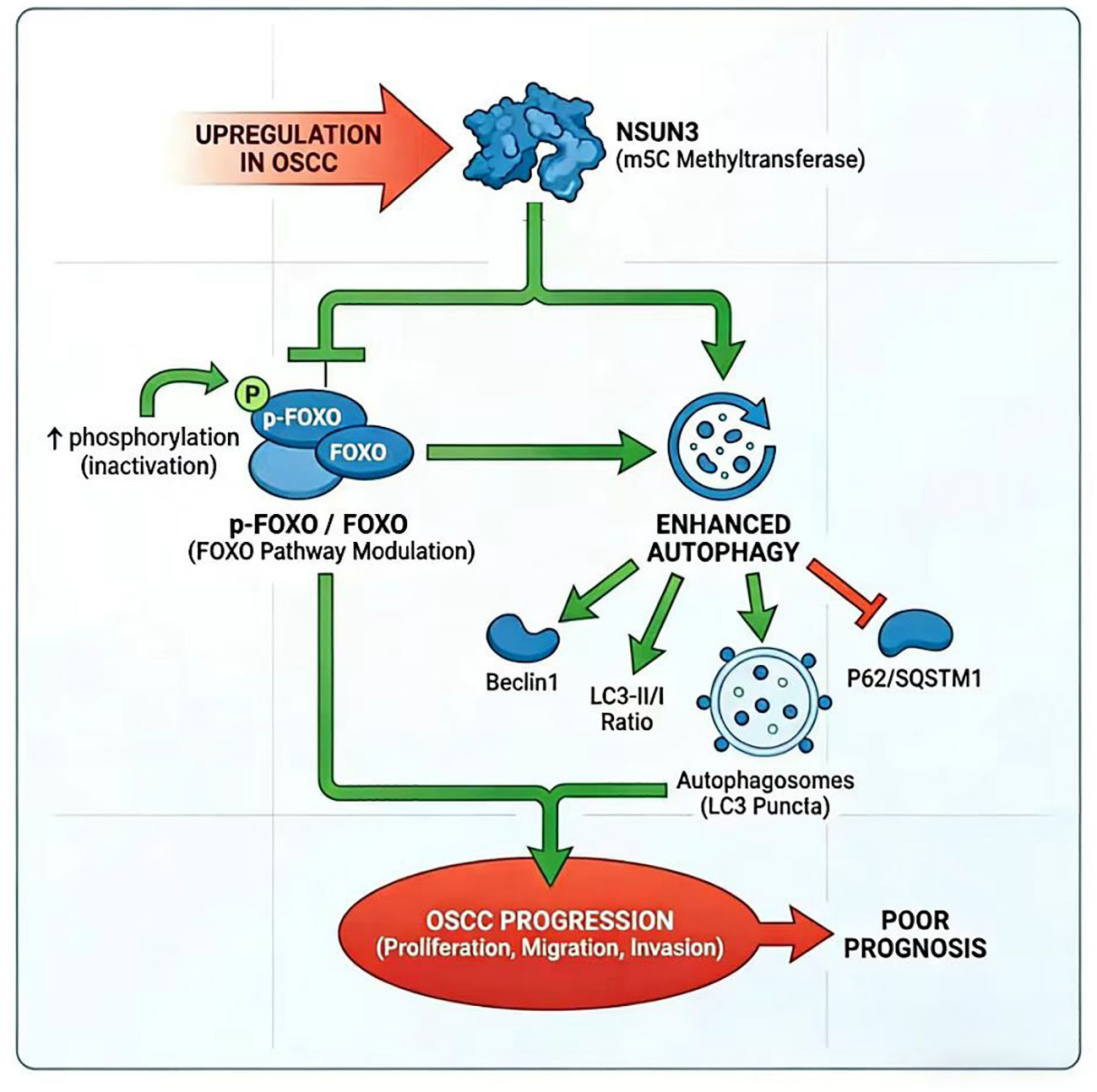
Schematic model illustrating that NSUN3 promotes OSCC progression by activating autophagy, which is potentially mediated by the modulation of the FOXO pathway.

In conclusion, our findings identified NSUN3 as a novel promoter of OSCC progression, a function which is likely mediated through the modulation of the FOXO/autophagy signaling axis. These results not only expanded the understanding of m^5^C RNA methylation in oral carcinogenesis but also suggested NSUN3 as a potential prognostic marker and therapeutic target in OSCC. Targeting the NSUN3−FOXO−autophagy axis offers a promising novel strategy for OSCC treatment, particularly in patients with high NSUN3 expression and aggressive disease. Based on these findings, we propose a working model illustrating how NSUN3 promotes OSCC progression by activating autophagy via modulation of the FOXO pathway (**Figure 6**).

## 5 Conclusion

This study demonstrated that NSUN3 was upregulated in OSCC and served as an independent prognostic factor for poor survival. Functionally, NSUN3 promoted OSCC cell proliferation, migration, and invasion. Mechanistically, NSUN3 knockdown suppressed autophagy and was associated with increased inhibitory phosphorylation of FOXO1 and FOXO3. Rescue experiments confirmed that autophagy contributes to NSUN3-mediated OSCC progression, although the partial restoration observed suggests the involvement of additional mechanisms. These findings highlight NSUN3 as a key regulator of OSCC progression and a potential therapeutic target worthy of further investigation.

## Data Availability

The datasets presented in this article are not readily available because due to legal and ethical considerations, the research data are not publicly available. Requests to access the datasets should be directed to wushuangjiang, wushuangjiang21@swmu.edu.cn.
