# Supplementary material for: NSUN3 promotes oral squamous cell carcinoma progression through autophagy activation and FOXO pathway modulation
Source: Front Oncol. 2026 Apr 22;16:1807220. doi: 10.3389/fonc.2026.1807220 (PMC13143738; doi:10.3389/fonc.2026.1807220)
Supplement: Supplementary file 1 [file Image1.pdf]

a

b

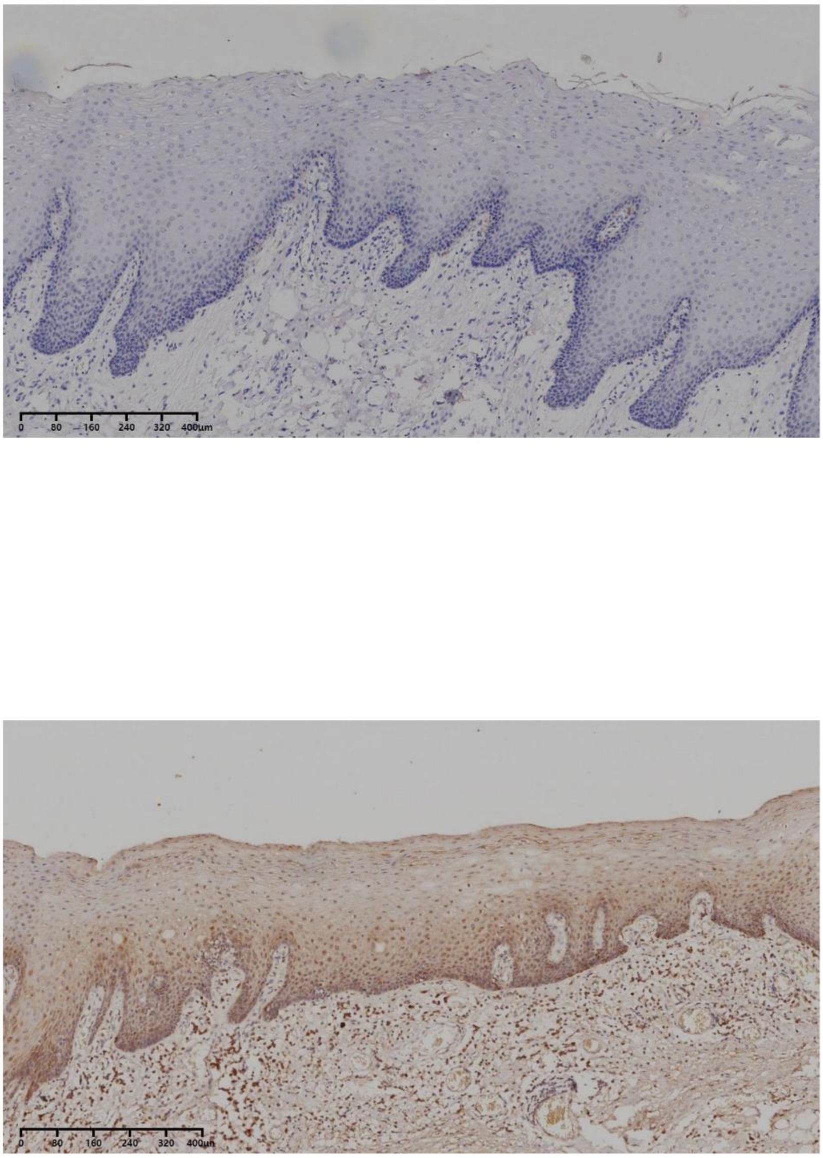

Supplementary Figure S1. Immunohistochemistry controls. **a** Negative control: adjacent normal oral mucosa incubated with PBS instead of the primary antibody, showing no detectable immunostaining. **b** Positive control: adjacent normal oral mucosa stained with anti-Ki-67 antibody, showing nuclear positivity
